# Exosomal RNA Expression Profiles and Their Prediction Performance in Patients With Gestational Diabetes Mellitus and Macrosomia

**DOI:** 10.3389/fendo.2022.864971

**Published:** 2022-04-25

**Authors:** Yingdi Yuan, Ying Li, Lingmin Hu, Juan Wen

**Affiliations:** ^1^ Department of Pediatrics, The First People’s Hospital of Lianyungang, Xuzhou Medical University Affiliated Hospital of Lianyungang (Lianyungang Clinical College of Nanjing Medical University), Lianyungang, China; ^2^ Department of Obstetrics and Gynecology, The Affiliated Wuxi People’s Hospital of Nanjing Medical University, Wuxi, China; ^3^ Department of Reproduction, Changzhou Maternal and Child Health Care Hospital, Changzhou Medical Center, Nanjing Medical University, Changzhou, China; ^4^ Nanjing Maternity and Child Health Care Institute, Women’s Hospital of Nanjing Medical University, Nanjing Maternity and Child Health Care Hospital, Nanjing, China

**Keywords:** exosomal RNA, gestational diabetes mellitus, macrosomia, ROC curves, graphical nomogram

## Abstract

**Introduction:**

Exosomes are cell-derived vesicles that are present in many biological fluids. Exosomal RNAs in cord blood may allow intercellular communication between mother and fetus. We aimed to establish exosomal RNA expression profiles in cord blood from patients with gestational diabetes mellitus and macrosomia (GDM-M) and evaluate their prediction performance.

**Methods:**

We used microarray technology to establish the differential messenger RNA (mRNA), long non-coding RNA (lncRNA), and circular RNA (circRNA) expression profiles in cord blood exosomes from 3 patients with GDM-M compared with 3 patients with GDM and normal neonatal weight, followed by qPCR validation in an additional 40 patients with GDM. Logistic regression, receiver operating characteristic (ROC) curves, and graphical nomogram were applied to evaluate the performance of exosomal RNA (in peripheral blood) in macrosomia prediction.

**Results:**

A total of 98 mRNAs, 372 lncRNAs, and 452 circRNAs were differentially expressed in cord blood exosomes from patients with GDM-M. Pathway analysis based on screening data showed that the differential genes were associated with Phosphatidylinositol 3'-kinase (PI3acK)-Akt signaling pathway, Janus kinase/signal transducers and activators of transcription (JAK/STAT) signaling pathway, Transforming growth factor (TGF)-beta signaling pathway, insulin resistance, glycerolipid metabolism, fatty acid degradation, and mammalian target of rapamycin (mTOR) signaling pathway. After validation by qPCR, the expressions of GDF3, PROM1, AC006064.4, lnc-HPS6-1:1, and circ_0014635 were significantly increased and the expression of lnc-ZFHX3-7:1 was significantly decreased in cord blood exosomes of an additional 20 patients with GDM-M. The risk prediction performance of the expression of these validated genes (in peripheral blood exosomes) for GDM-related macrosomia was also evaluated. Only GDF3 expression and AC006064.4 expression showed well prediction performance [area under the curve (AUC) = 0.78 and 0.74, respectively]. Excitingly, the model including maternal age, fasting plasma glucose, 2-h plasma glucose, GDF3 expression, and AC006064.4 expression in peripheral blood exosomes had better prediction performance with an AUC of 0.86 (95% CI = 0.75–0.97).

**Conclusion:**

These results showed that exosomal RNAs are aberrantly expressed in the cord blood of patients with GDM-M and highlighted the importance of exosomal RNAs in peripheral blood for GDM-M prediction.

## Introduction

Macrosomia is defined as babies with birth weight of 4,000 g or more and is the most common adverse pregnancy outcome of gestational diabetes mellitus (GDM) (1). The latest statistics show that more than 16.7% of live births worldwide are affected by hyperglycemia during pregnancy (2). In recent years, with the improvement of China’s living standards and the implementation of the “universal two-child” policy, there are a growing number of overweight or elderly pregnant women and the incidence of GDM is increasing by years, resulting in a sharp increase in the incidence of GDM-related macrosomia (3), posing a serious threat to maternal and infant health. GDM-related macrosomia is mostly metabolic asymmetries, manifested by a significant increase in body fat, while the volume of other tissues/organs does not increase significantly, which not only affects the growth and development of infants but also greatly increases the risk of obesity or diabetes in their future life (1). According to the Developmental Origins of Health and Disease (DOHaD) theory, the nutrition and nurturing environment in the first 1,000 days of life (such as intrauterine hyperglycemia) are crucial to an individual’s lifetime health (4). Therefore, revealing the pathogenesis of GDM-related macrosomia is of great significance for controlling macrosomia occurrence, reducing maternal and infant complications, and delaying the occurrence of obesity or diabetes in offspring of patients with GDM.

Exosomes are a kind of vesicles with a diameter of 30–150 nm, which are important carriers of intercellular communication (5). Cells secrete exosomes after receiving certain stimuli, and exosomes can enter recipient cells through paracrine secretion, membrane fusion, ligand-receptor binding, and phagocytosis to release numerous bioactive substances wrapped, such as messenger RNA (mRNA), non-coding RNA, and protein, which can affect the biological functions of recipient cells, and then participate in various physiological and pathological processes (5, 6).

Studies have shown that placenta-derived exosomes can be detected in maternal circulation as early as 6 weeks of gestation, and the concentration of placental exosomes increases with the progression of pregnancy (7). In women with GDM in the early, middle, and late stages of pregnancy, the concentration of placental exosomes is significantly higher than that of normal pregnant women at the same stage (7), suggesting that the process of releasing exosomes from placenta to circulation may be affected by blood glucose. As exosomes have a double-layer membrane that protects them from degradation, they can be widely detected in various body fluids such as peripheral blood, cord blood, amniotic fluid, milk, and saliva and can be used as biomarkers for the occurrence and development of diseases (6, 8). Exosomal RNAs, such as mRNA, long non-coding RNA (lncRNA), and circular RNA (circRNA), were reported to play an important role in the pathogenesis and diagnosis of diseases (9). For example, exosomal lncRNA-KLF3-AS1 derived from human mesenchymal stem cells (hMSCs) suppressed Interleukin (IL)-1β-induced apoptosis of chondrocytes (10). Moreover, in our previous study, we found that exosomal mRNA, lncRNA, and circRNA were aberrantly expressed in the cord blood of patients with GDM (11, 12). However, exosomal RNAs involved in the occurrence of GDM-related macrosomia and exosomal RNAs as biomarkers for macrosomia have not been reported.

Considering that exosomal RNAs may play an important role in GDM-related macrosomia, we used microarray technology to establish the exosomal mRNA, lncRNA, and circRNA expression profiles in cord blood of patients with GDM and macrosomia (GDM-M). Moreover, a risk prediction model including maternal blood glucose and expression level of exosomal RNAs in peripheral blood was constructed for GDM-related macrosomia by logistic regression.

## Methods

### Ethics Statement

This study was approved by the ethics committee of the Women’s Hospital of Nanjing Medical University (NFKSL2018-107), and all subjects gave a written informed consent.

### Patients and Sample Collection

Participants were recruited at the Changzhou Maternity and Child Health Care Hospital and Wuxi People’s Hospital from July 2019 to June 2021. GDM was diagnosed by 75 g oral glucose tolerance test performed between 24 and 28 gestational weeks (fasting blood glucose ≥5.1 mmol/L, 1-h blood glucose ≥10.0 mmol/L, or 2-h blood glucose ≥8.5 mmol/L) (13), and macrosomia was defined as birth weight more than 4,000 g. The patients with GDM-M were assigned to the case group, while patients with GDM and normal neonatal weight (2,500 g ≤ birth weight < 4,000 g) were assigned to the control group. Participants diagnosed with diabetes before pregnancy were excluded from this study. A total of 46 umbilical cord blood samples were collected from the umbilical vein immediately after delivery of the fetus during Cesarean section (23 patients with GDM-M and 23 controls) according to the standard operating procedure. All participants were divided into two sets, 6 participants (3 patients with GDM-M and 3 controls) for microarray screening and 40 participants (20 patients with GDM-M and 20 controls) for validation. For validation set, peripheral blood between 24 and 28 gestational weeks was additionally discharged from the sample bank for prediction test.

### Exosomal RNA Extraction and Microarray Analysis

The methods for exosomal RNA extraction and microarray analysis have been previously described (11, 12, 14, 15). Exosomes were prepared from the umbilical cord blood/peripheral blood. Briefly, cord blood/peripheral blood was centrifuged at 3,000 g for 15 min at 4°C. Supernatants were then centrifuged at 12,000 g for 30 min at 4°C. Then, supernatants were filtered through 0.45-μm polyvinylidene fluoride (PVDF) membrane and isolated in a final ultracentrifugation at 100,000 g for 180 min at 4°C. The exosome pellet was resuspended in PBS or lysis buffer. The resulting exosomes were next analyzed with the Nanosight Nano ZS device (Malvern Instruments, UK). Total exosomal RNA was extracted using Serum/Plasma Kit (QIAGEN, Germany). Total RNA was amplified and labeled by Low Input Quick Amp Labeling Kit (Agilent Technologies, USA). Each slide was hybridized with 1.65 μg Cy3-labeled cRNA using Gene Expression Hybridization Kit (Agilent Technologies, USA) in Hybridization Oven (Agilent Technologies, USA). After 17 h of hybridization, slides were washed in staining dishes (Thermo Shandon, USA) with Gene Expression Wash Buffer Kit (Agilent Technologies, USA). Slides were scanned by Agilent Microarray Scanner (Agilent Technologies, USA) with default settings. Data were extracted with Feature Extraction software 12.0 (Agilent Technologies, USA). Limma packages in R (version: 3.26.9) was used to conduct the background correction and the normalization of the raw data.

### Quantitative Real-Time PCR

Complementary DNA was acquired from reverse transcription of 500 ng RNA using PrimeScript RT reagent with gDNA Eraser (TaKaRa, Japan). Primer-BLAST was applied to design the specific primers, which were synthesized by Realgene (Nanjing, China). After the optimal annealing temperatures were determined, quantitative real-time PCR (qPCR) was performed on the Life Tech-ViiA7 system (Applied Biosystems, USA) using PowerUP SYBR Green Master Mix (Applied Biosystems, USA) to measure the relative expression level of each mRNA/lncRNA/circRNA. Glyceraldehyde phosphate dehydrogenase (GAPDH) was used as internal control, and the relative expression level of each mRNA/lncRNA/circRNA was calculated with the 2^-△△Ct^ method (16).

### Functional Enrichment Analyses

DAVID Bioinformatics Resources 6.8 was used to analyze the associated gene functions of the differentially expressed lncRNAs and the parental gene functions of the differentially expressed circRNAs (17). Gene Ontology (GO) analysis was conducted based on biological processes, cellular components, and molecular functions, and Kyoto Encyclopedia of Genes and Genomes (KEGG) was used to analyze the related biological pathways. The -log (P-value) was used as enrichment score to indicate the significance of the correlation.

### Annotation for lncRNA/miRNA and circRNA/miRNA Interactions

MicroRNAs (miRNAs) are a class of endogenous, non-coding, single-stranded RNAs with a length of approximately 22 nucleotides that regulate many cell processes (18). A large amount of evidence has indicated that exosomal lncRNAs and circRNAs could act as competing endogenous RNA (ceRNA) molecules or efficient miRNA sponges to regulate miRNA-targeted gene expression, transcription, and protein synthesis (19, 20). The miRanda (v3.3a) was used to predict lncRNA/miRNA and circRNA/miRNA interactions (21). With the database, we searched miRNA response elements (MREs) on lncRNAs/circRNAs and selected miRNAs based on the seed-match sequences.

### Statistical Analyses

All data are expressed as mean ± standard deviation (SD). The differences between groups were evaluated with *t*-test after normality test. Risk prediction models to classify the patients with GDM-M and controls were constructed using logistic regression. The model performance was evaluated by receiver operating characteristic (ROC) curves, and the area under the curve (AUC) was used to classify the patients with GDM-M and controls. The sensitivity, specificity, negative predictive value (NPV), and positive predictive value (PPV) were calculated to illustrate the model effects using the “best threshold” criteria of the ROC curve. A graphical nomogram was also produced for the constructed model so that the individual-specific risk of macrosomia from patients with GDM could be easily approximated (22). All the statistical analyses were performed with R software (version 3.3.0), and *P* ≤ 0.05 was considered statistically significant.

## Results

### Participant Characteristics

The clinic data for the participants are shown in **Table 1**. We compared maternal characteristics between patients with GDM-M and controls. For the validation set, we found maternal age, fasting plasma glucose, and 2-h plasma glucose were significantly higher in patients with GDM-M (*P* < 0.05).

**Table 1:** Clinical data for the patients with GDM-M and controls.

Variables	Microarray screening set			Validation set		
	GDM-M (n = 3)	Controls (n = 3)	*P*	GDM-M (n = 20)	Controls (n = 20)	*P*
Maternal age (years)	32.0 ± 3.6	28.7 ± 2.5	0.259	30.8 ± 3.5	28.2 ± 4.1	0.034
Gestational week	39.7 ± 1.2	39.0 ± 1.0	0.492	39.6 ± 1.0	39.1 ± 1.1	0.121
Neonatal weight (g)	4,160.0 ± 165.2	2,950.0 ± 312.2	0.004	4,101.0 ± 72.4	3,055.5 ± 336.0	<0.001
Neonatal body fat (g)	937.7 ± 94.1	697.8 ± 57.7	0.020	904.2 ± 110.9	788.6 ± 139.1	0.006
Fasting plasma glucose/mmol/L	5.5 ± 0.3	5.3 ± 0.1	0.313	5.6 ± 0.4	5.4 ± 0.3	0.032
1-h plasma glucose/mmol/L	11.1 ± 0.3	10.1 ± 0.8	0.108	10.5 ± 0.6	10.3 ± 0.4	0.150
2-h plasma glucose/mmol/L	10.4 ± 0.2	9.1 ± 0.1	0.001	9.3 ± 0.6	8.8 ± 0.5	0.006

GDM-M, gestational diabetes mellitus and macrosomia.

### RNA Expression Profiles in Cord Blood Exosomes From Patients With GDM-M and Controls

**Figure 1:**
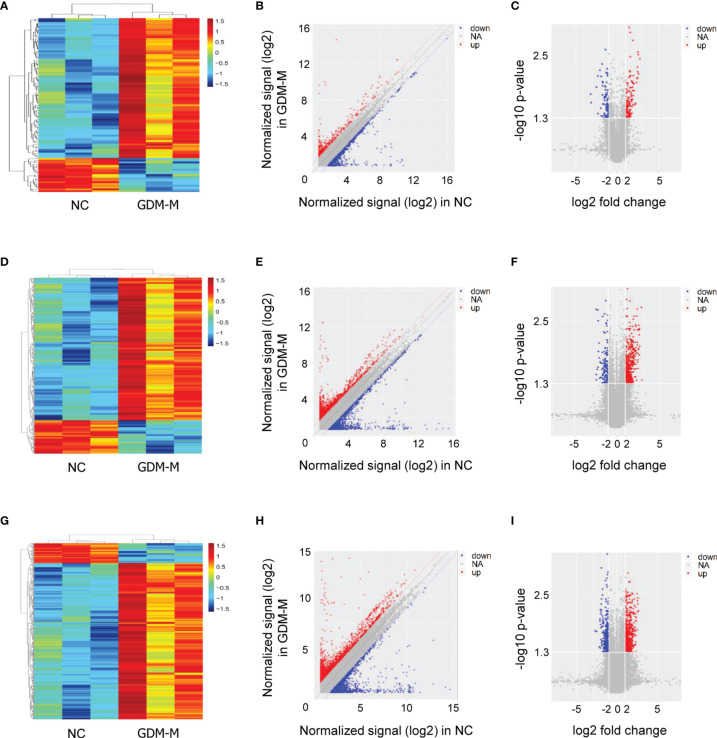
Exosomal RNA expression profiles in cord blood between patients with GDM-M and controls (NC). **(A–C)** Cluster analysis, scatter plots, and volcano plots for mRNA. **(D–F)** Cluster analysis, scatter plots, and volcano plots for lncRNA. **(G–I)** Cluster analysis, scatter plots, and volcano plots for circRNA. GDM-M, gestational diabetes mellitus and macrosomia; mRNA, messenger RNA; lncRNA, long non-coding RNA; circRNA, circular RNA.

According to the microarray data, heat map, scatter plot, and volcano plot were used to display the differentially expressed RNAs (mRNAs, lncRNAs, and circRNAs) in cord blood exosomes between patients with GDM-M and controls (**Figure 1**). In total, 18,263 mRNAs, 58,539 lncRNAs, and 88,371 circRNAs were detected in cord blood exosomes. Of these, 98 mRNAs, 372 lncRNAs, and 452 circRNAs were differentially expressed in patients with GDM-M according to the criteria of fold change (FC) ≥ |2.0| and *P* < 0.05 (**Tables S1–S3**).

**Figure 2:**
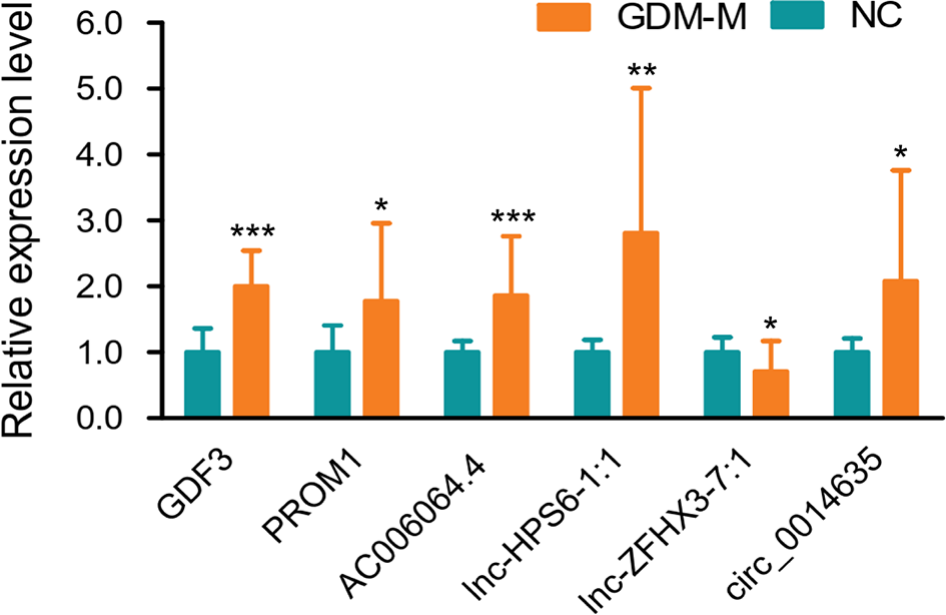
Validation for differentially expressed mRNAs, lncRNAs, and circRNA by qPCR. * *P* < 0.01, ** *P* < 0.01, and *** *P* < 0.001. GDM-M, gestational diabetes mellitus and macrosomia; mRNA, messenger RNA; lncRNA, long non-coding RNA; circRNA, circular RNA.

Based on FC ≥ |3.0|, *P* < 0.001, and relatively high abundance, we then selected 5 mRNAs (NUTM1, GDF3, C17orf105, PROM1, and NFE2L1), 10 lncRNAs (AC073912.1, IL10RB-DT:19, lnc-FAM49B-4:1, lnc-ZFHX3-7:1, AC006064.4, lnc-HPS6-1:1, lnc-SEC11A-2:1, lnc-CAPZA3-3:2, lnc-TNFSF13B-2:1, and lnc-SOX6-1:1), and 10 circRNAs (circ_0012756, circ_0041183, circ_0065086, circ_0014635, circ_0075695, circ_0074153, circ_0068824, circ_0048469, circ_0066837, and circ_0026688) and validated their expression in cord blood exosomes from 20 patients with GDM-M and 20 controls. Confirming microarray data, qPCR results showed that the expressions of GDF3, PROM1, AC006064.4, lnc-HPS6-1:1, and circ_0014635 were increased and the expression of lnc-ZFHX3-7:1 was decreased in patients with GDM-M (**Figure 2**).

### Functional Enrichment Analyses of the Differentially Expressed Genes

Cytoscape (3.8.0) was firstly applied to visualize pathway information of the differentially expressed mRNAs. The results showed that these genes were associated with PI3K-Akt signaling pathway, JAK-STAT signaling pathway, and TGF-beta signaling pathway (**Figure S1**). Differentially expressed lncRNAs were chosen to predict target mRNAs *via cis-* or *trans-*regulatory effects (23). To examine the potential function of mRNAs related to differentially expressed lncRNAs, GO analysis and KEGG pathway analysis were performed. In the GO analysis, the most significantly enriched term for biological processes, cellular components, and molecular functions was macromolecule localization, cytoplasm, and nucleotide binding, respectively (**Figure S2**). KEGG results indicated that the differentially expressed lncRNAs were involved in lysosome, insulin resistance, lipoic acid metabolism, glycerolipid metabolism, fatty acid degradation, protein processing in endoplasmic reticulum, and amino sugar and nucleotide sugar metabolism (**Figure S3**). Then, we used these pathways to construct a pathway network to investigate the key pathways associated with macrosomia in women with GDM. As shown in **Figure S4**, the exchanges with these pathways largely depended on the existence of citrate cycle and mTOR signaling pathway. Each circle represents a pathway, and the larger the circle, the more pathways it interacts with, indicating that its core position in the network is more important.

For the differential circRNA parental genes, the results of GO analysis indicated that the most significantly enriched term for biological processes, cellular components, and molecular functions was establishment of localization in cell, cell junction, and extracellular matrix structural constituent, respectively (**Figure S5**). KEGG results showed that the differential circRNA was involved in focal adhesion, regulation of actin cytoskeleton, long-term potentiation, relaxin signaling pathway, and glutamatergic synapse (**Figure S6**). Moreover, the exchanges with these pathways largely depended on the existence of the regulation of actin cytoskeleton and Vascular endothelial growth factor (VEGF) signaling pathway (**Figure S7**).

### Prediction of lncRNA/miRNA and circRNA/miRNA Interactions

Interactions between the differential lncRNAs/circRNAs and miRNAs were theoretically predicted by miRanda based on the MREs. We found 1,302 miRNAs paired with 211 differentially expressed lncRNAs and 1,814 miRNAs paired with 438 differentially expressed lncRNAs with the criteria of max score ≥150 and max energy ≤-25 (**Table S4, S5**); the lower the max energy is, the more significant the correlation.

### Prediction Models Constructed for GDM-Related Macrosomia

**Figure 3:**
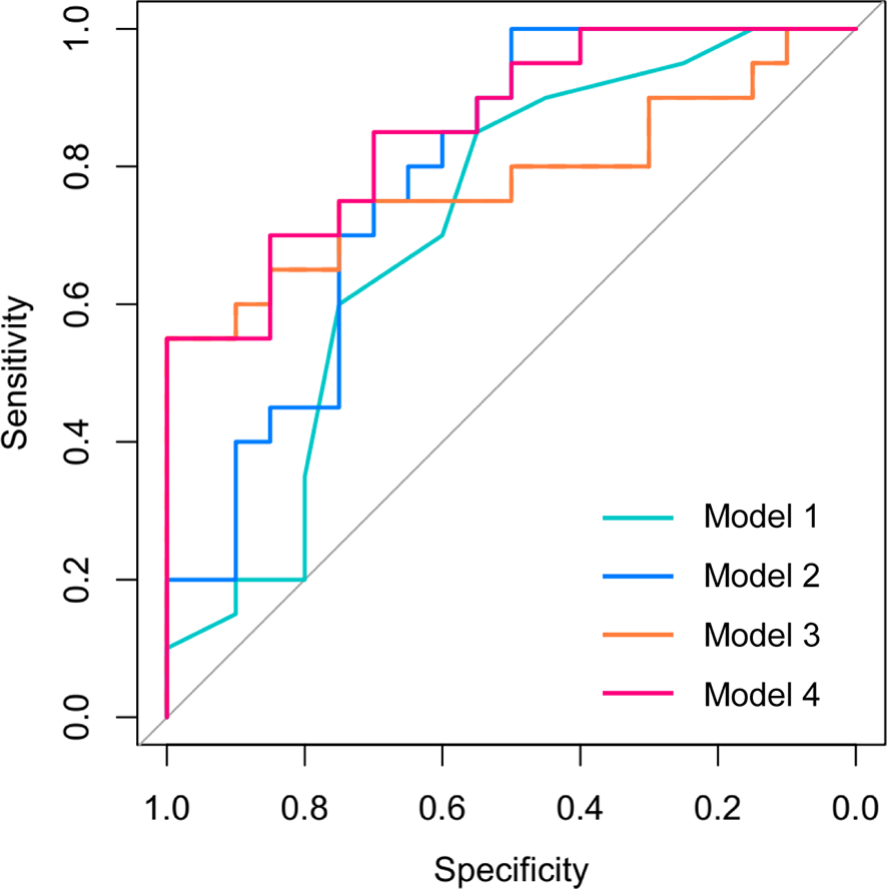
Receiver operating characteristic (ROC) curves for the logistic regression model in the prediction of macrosomia in patients with GDM. Model 1 included maternal age. Model 2 included maternal age, fasting plasma glucose, and 2-h plasma glucose. Model 3 included GDF3 expression and AC006064.4 expression in peripheral blood exosomes. Model 4 included maternal age, fasting plasma glucose, 2-h plasma glucose, GDF3 expression, and AC006064.4 expression in peripheral blood exosomes.

Then, we analyzed the expression of the differential mRNAs, lncRNAs, and circRNAs (GDF3, PROM1, AC006064.4, lnc-HPS6-1:1, lnc-ZFHX3-7:1, and circ_0014635) in exosomes of peripheral blood at 24–28 gestational weeks. ROC curves were constructed to evaluate the risk prediction performance of the expression of these validated genes for GDM-related macrosomia. Only GDF3 expression and AC006064.4 expression in peripheral blood exosomes showed well prediction performance (GDF3 expression: AUC = 0.78, 95% CI = 0.63–0.94, *P* = 0.002; AC006064.4 expression: AUC = 0.74, 95% CI = 0.58–0.90, *P* = 0.009) (**Figure S8**). Results of logistic regression also showed that the expressions of GDF3 and AC006064.4 in peripheral blood exosomes were associated with increased risk of GDM-M (GDF3: OR = 3.82, 95% CI = 1.06–13.83; AC006064.4: OR = 3.3, 95% CI = 1.10–9.93). Therefore, maternal age, fasting plasma glucose, 2-h plasma glucose, GDF3 expression, and AC006064.4 expression in peripheral blood exosomes were considered as predictors for macrosomia. Then, we analyzed the discriminative ability of these predictors in different combinations (**Figure 3**). As shown in **Table 2**, the AUC of Model 1 (maternal age), Model 2 (maternal age + fasting plasma glucose + 2-h plasma glucose), and Model 3 (GDF3 expression + AC006064.4 expression) was 0.71, 0.79, and 0.78, respectively. In fact, the AUC of Model 4 (maternal age + fasting plasma glucose + 2-h plasma glucose + GDF3 expression + AC006064.4 expression) was even better than the above models at 0.86. For Model 4, the Hosmer–Lemeshow χ^2^ was 3.98 (degrees of freedom = 8 and *P* = 0.859), giving no cause for concern over model fit or calibration.

**Table 2:** The discriminative ability of four panels between patients with GDM-M and controls.

Model		AUC	95% CI of AUC	Threshold	Specificity	Sensitivity	NPV	PPV
Model 1	Maternal age	0.71	0.55–0.88	0.41	0.55	0.85	0.79	0.65
Model 2	Maternal age + fasting plasma glucose + 2-h plasma glucose	0.79	0.65–0.93	0.26	0.50	1.00	1.00	0.67
Model 3	GDF3 expression + AC006064.4 expression (in peripheral blood exosomes)	0.78	0.63–0.93	0.69	1.00	0.55	0.69	1.00
Model 4	Maternal age + fasting plasma glucose + 2-h plasma glucose + GDF3 expression + AC006064.4 expression (in peripheral blood exosomes)	0.86	0.75–0.97	0.76	1.00	0.55	0.69	1.00

GDM-M, gestational diabetes mellitus and macrosomia; AUC, area under the curve; NPV, negative predictive value; PPV, positive predictive value.

**Figure 4:**
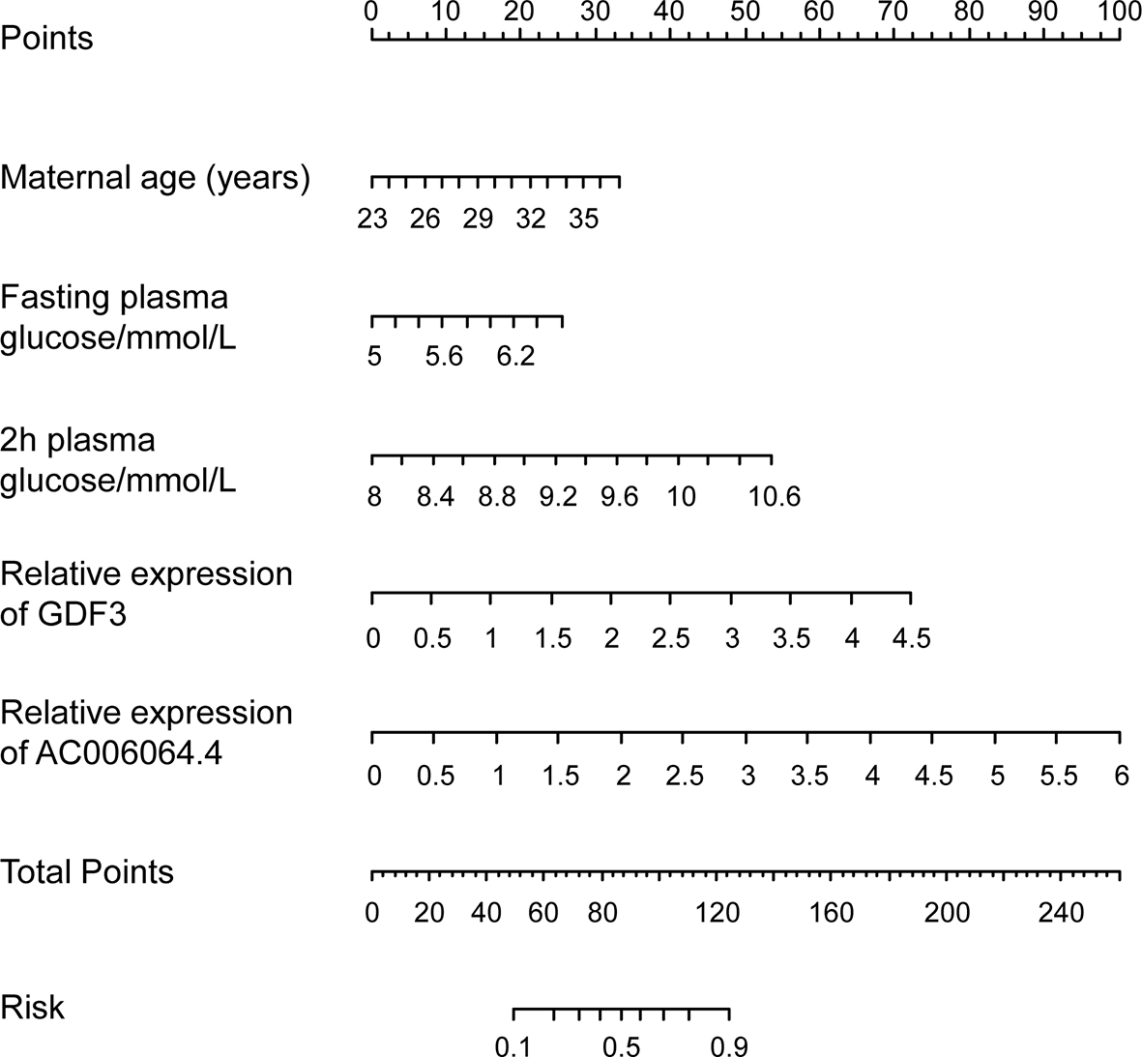
Predictive graphical nomogram for macrosomia risk from patients with GDM. Predictors included maternal age, fasting plasma glucose, 2-h plasma glucose, GDF3 expression, and AC006064.4 expression in peripheral blood exosomes. GDM, gestational diabetes mellitus.

Moreover, the graphical nomogram for Model 4 is presented in **Figure 4**. Each patient characteristic is aligned with the corresponding number of points on the uppermost point scale. After all characteristics are considered, the user sums all points and aligns the sum on the “total points” line with the predicted risk of macrosomia from patients with GDM.

## Discussion

The pathogenesis of GDM-induced macrosomia is very complex, involving many factors such as heredity, nutrition, and endocrine (1). Previous studies have suggested that high levels of glucose in the GDM mother can directly enter the fetus without being obstructed by the placental barrier. Long-term hyperglycemia in the fetus can stimulate islet cell proliferation, promote insulin secretion, cause hyperinsulinemia, and then promote protein synthesis and inhibit lipolysis, leading to macrosomia (1). However, after strict blood glucose control in pregnant women with GDM, the incidence of macrosomia has not been significantly reduced (24), indicating that the mechanism of macrosomia induced by GDM is particularly complex. Recent studies have shown that the high glucose environment in GDM mothers can stimulate the secretion of various cytokines (such as insulin-like growth factor (IGF)-1/2, leptin, adiponectin, etc.) from the placenta into the fetus *via* umbilical vein and, in combination with high glucose *in utero*, stimulate the synthesis of fat in the fetus, thus causing fetal overgrowth (25–28). Even so, current studies cannot fully elucidate the molecular mechanism of macrosomia induced by GDM, suggesting that there may be other mechanisms involved in the communication between placenta and fetus stimulated by maternal high glucose, thus promoting macrosomia. Exosomes are endosome-derived membrane vesicles that contain numerous RNAs and allow intercellular communication (5). Based on cord blood exosomes, this study found that the expression of GDF3, PROM1, AC006064.4, lnc-HPS6-1:1, and circ_0014635 were significantly increased, and the expression of lnc-ZFHX3-7:1 was significantly decreased in cord blood exosomes of patients with GDM-M, which will help to understand the pathogenesis of GDM-related macrosomia.

Pathway analysis based on screening data showed that the differentially expressed exosomal RNAs were associated with PI3K-Akt signaling pathway, JAK-STAT signaling pathway, TGF-beta signaling pathway, insulin resistance, glycerolipid metabolism, fatty acid degradation, and mTOR signaling pathway, which are important in GDM development and fetus growth. For example, the activation of the PI3K-Akt pathway has been shown to be important in promoting placenta development and fetal growth in both humans and rodents (29, 30). Insulin resistance, glycerolipid metabolism, and fatty acid degradation are primary biological processes in metabolic diseases. These metabolic alterations may contribute to the pathophysiology of GDM and may also influence fetal growth (31). Together, these altered exosomal RNAs are associated with metabolic and growth-related signaling pathways.

LncRNA and circRNA in exosomes can exert ceRNA activity in the cytoplasm and interfere with the transcriptional regulation of target genes by miRNA (20, 32, 33). In this study, through lncRNA/miRNA and circRNA/miRNA interactions analysis, we found that most of the exosomal lncRNAs and circRNAs altered in the GDM-M group harbored miRNA binding sites, and some miRNAs were associated with GDM development and fetus growth. A previous miRNA microarray analysis had identified differential miRNA expression in placental tissues of normal controls and women with GDM (34), some of which could be found in our interaction analysis, such as miR-508, miR-27a, miR-137, miR-92a, miR-362-5p, and miR-502-5p. Therefore, we speculate that the roles of exosomal lncRNAs and circRNAs in GDM-M may be related to miRNA-mediated effects. The underlying mechanism of the lncRNA/circRNA–miRNA–target gene interaction in GDM-related macrosomia warrants further research.

Moreover, we constructed ROC curves and graphical nomogram to evaluate the risk prediction performance of the expression of the validated genes for GDM-related macrosomia and found that GDF3 expression and AC006064.4 expression in peripheral blood exosomes had good prediction performance. GDF3 (growth/differentiation factor 3), as a key regulatory gene of adipose differentiation (35), can regulate fat differentiation in high-fat diet mice through ALK7/Cripto receptor complex (36). Mice with high expression of GDF3 experienced a significant increase in body fat and hypertrophy of adipocytes in the high-fat diet (37). For AC006064.4, there are no relevant studies reported at present. Bioinformatics analysis showed that *has*-AC006064.4 was 422 bp in length and located at 12p13.31. It was highly homologous in human, mouse, and other mammals, and the transcription status of AC006064.4 included in the chromatin region was very active. To some extent, lncRNA AC006064.4 may have important biological functions. Further studies are needed to explore the function and mechanism of these exosomal RNAs in GDM development and fetal growth. By combining different predictors, we constructed four risk prediction models for GDM-related macrosomia. Model including maternal age, fasting plasma glucose, 2-h plasma glucose, GDF3 expression, and AC006064.4 expression in peripheral blood exosomes had well prediction performance with AUC of 0.86. To the best of our knowledge, this is the first study constructing prediction models and estimating sensitivity and specificity for GDM-related macrosomia. The model and its graphical nomogram provide an individual risk of macrosomia occurrence from pregnant women with GDM.

## Conclusions

Our results showed that exosomal RNAs are aberrantly expressed in the cord blood of patients with GDM-M and highlighted the importance of exosomal RNAs in predicting GDM-related macrosomia. This study also provides a basis for further studies on function and mechanism of exosomal mRNAs, lncRNAs, and circRNAs in GDM-related macrosomia. However, several limitations ought to be acknowledged. The worse glycemic control and advanced maternal age are known risk factors for macrosomia, and the two factors may be key confounders when considering potential pathophysiological links between exosomal RNA and macrosomia. Therefore, the uneven glycemic control and maternal age between the GDM-M group and the control group were two of the major limitations of this study. Moreover, experiments showing exact regulation mechanism of exosomal RNAs are lacking, and miRNA expression in peripheral blood and adipose tissue was not measured based on our lncRNA/miRNA and circRNA/miRNA interaction analysis. Further studies on function and mechanism of exosomal RNAs in GDM-M occurrence are warranted to validate and extend our findings. In addition, larger cohort studies with strict inclusion criteria are needed to demonstrate that exosomal RNAs are clinically applicable biomarkers.

## Data Availability Statement

The original contributions presented in the study are included in the article/**Supplementary Material**. Further inquiries can be directed to the corresponding authors.

## Ethics Statement

The studies involving human participants were reviewed and approved by the ethics committee of the Women’s Hospital of Nanjing Medical University (NFKSL2018-107). The patients/participants provided their written informed consent to participate in this study.

## Author Contributions

JW initiated, conceived, and supervised the study. LH and YL did data collection and performed the data analysis. YY did molecular biology experiment. All authors approved the final format of the submitted article.

## Funding

This work was supported by the Jiangsu Provincial Key Research and Development Program (BE2020626) and the National Natural Science Foundation of China (81900772).

## Conflict of Interest

The authors declare that the research was conducted in the absence of any commercial or financial relationships that could be construed as a potential conflict of interest.

## Publisher’s Note

All claims expressed in this article are solely those of the authors and do not necessarily represent those of their affiliated organizations, or those of the publisher, the editors and the reviewers. Any product that may be evaluated in this article, or claim that may be made by its manufacturer, is not guaranteed or endorsed by the publisher.
