# Supplementary material for: Exosomal RNA Expression Profiles and Their Prediction Performance in Patients With Gestational Diabetes Mellitus and Macrosomia
Source: Front Endocrinol (Lausanne). 2022 Apr 25;13:864971. doi: 10.3389/fendo.2022.864971 (PMC9082313; doi:10.3389/fendo.2022.864971)
Supplement: Supplementary file 1 [file DataSheet_1.docx]

**Supplementary Figures**


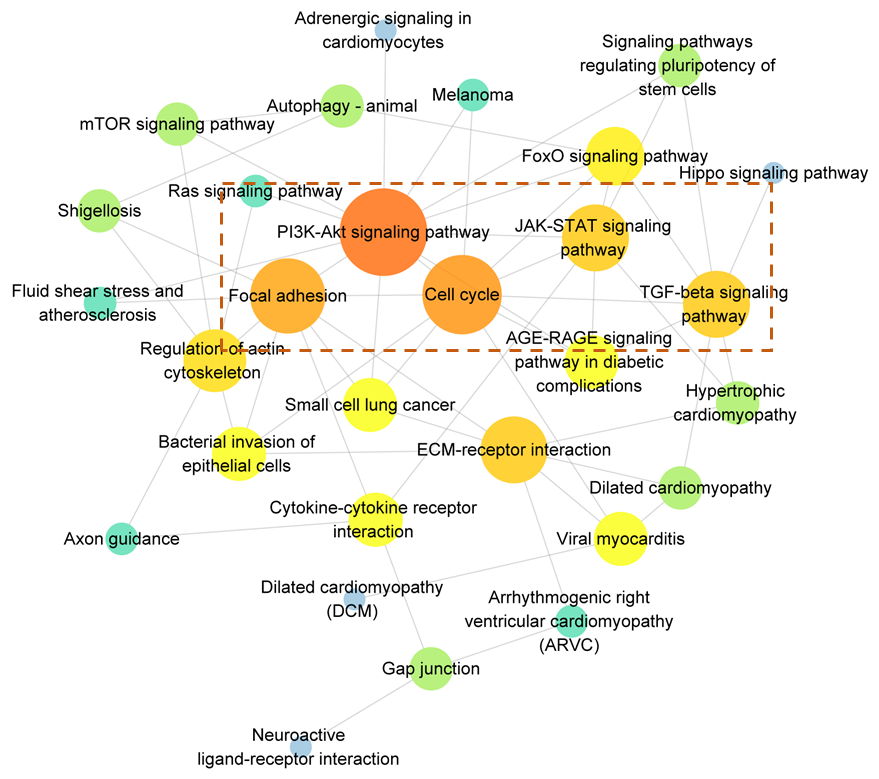


**Figure S1. Functional enrichment analysis of mRNAs.**


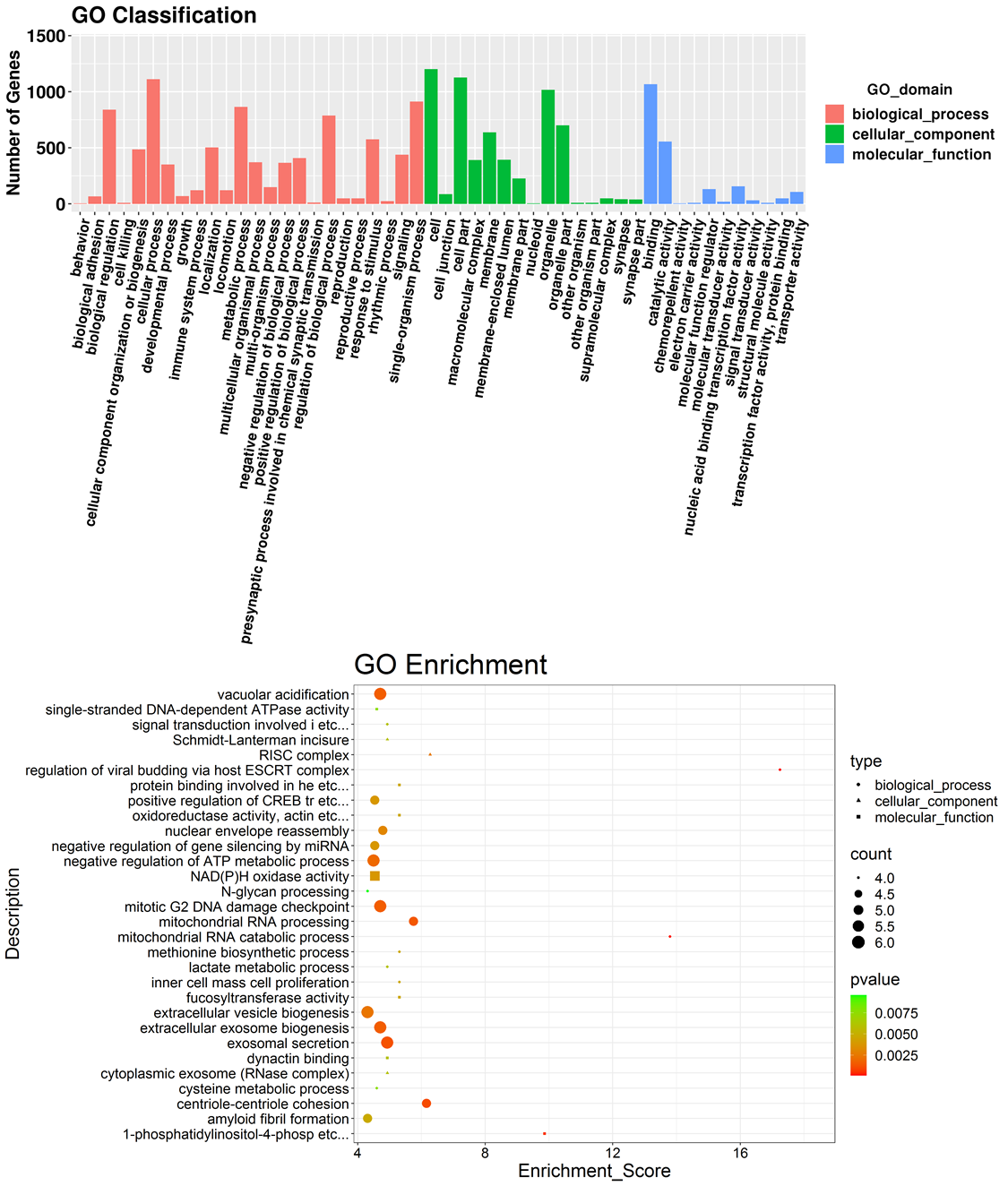


**Figure S2. GO analysis of differential lncRNA-associated mRNAs.**


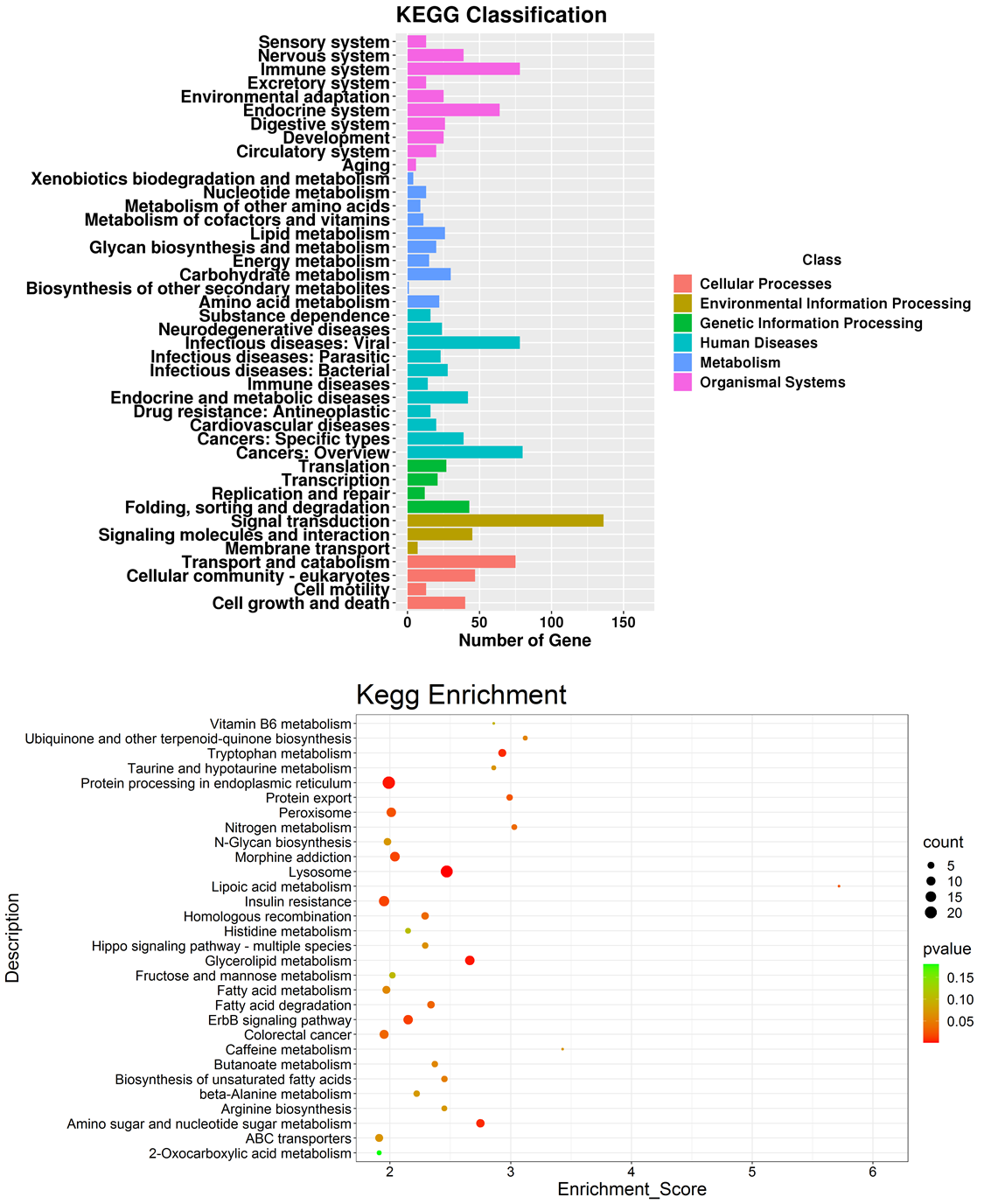


**Figure S3. KEGG analysis of differential lncRNA-associated mRNAs.**


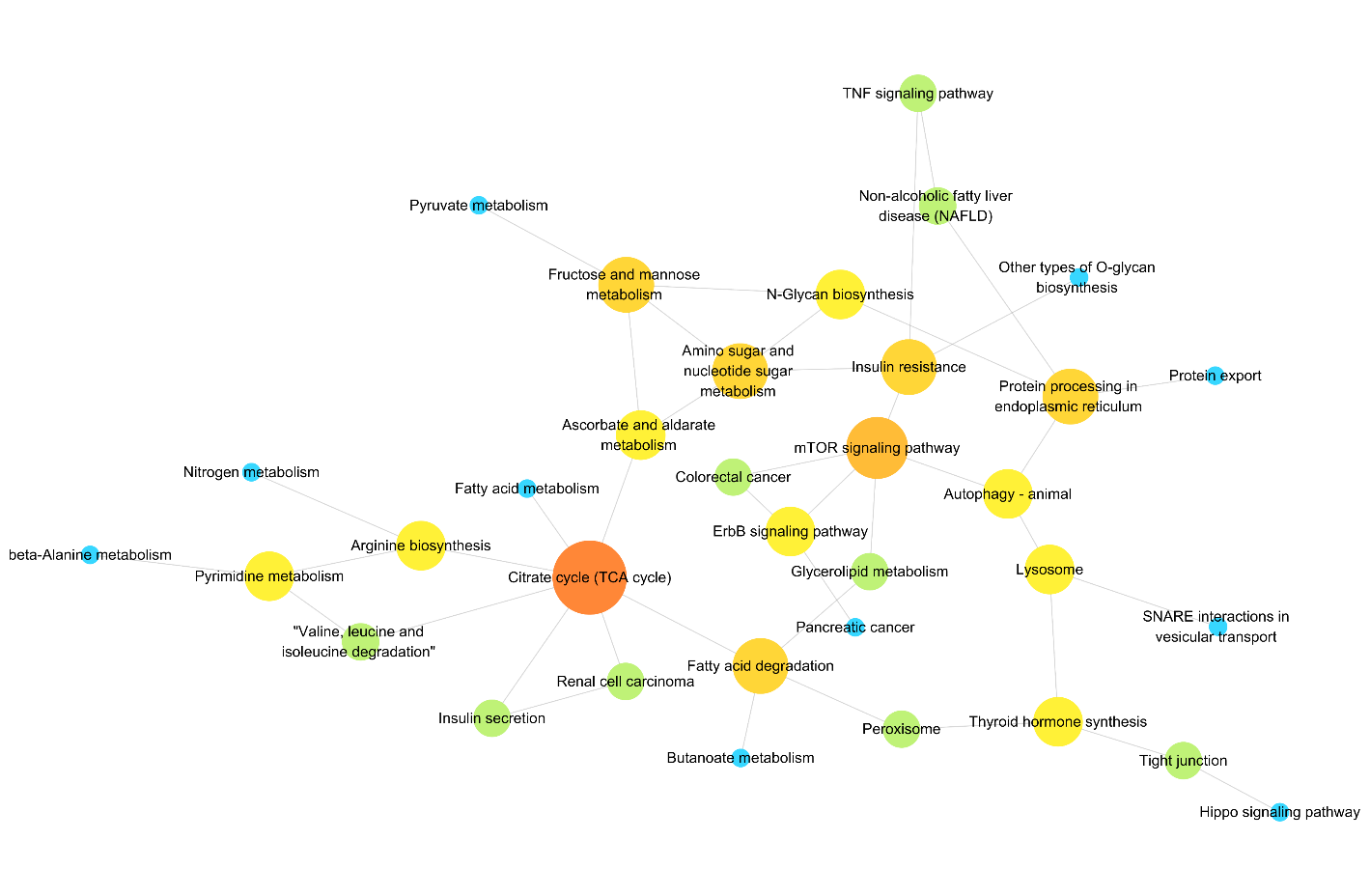


**Figure S4. Functional enrichment analysis of differential lncRNA-associated genes.**

**
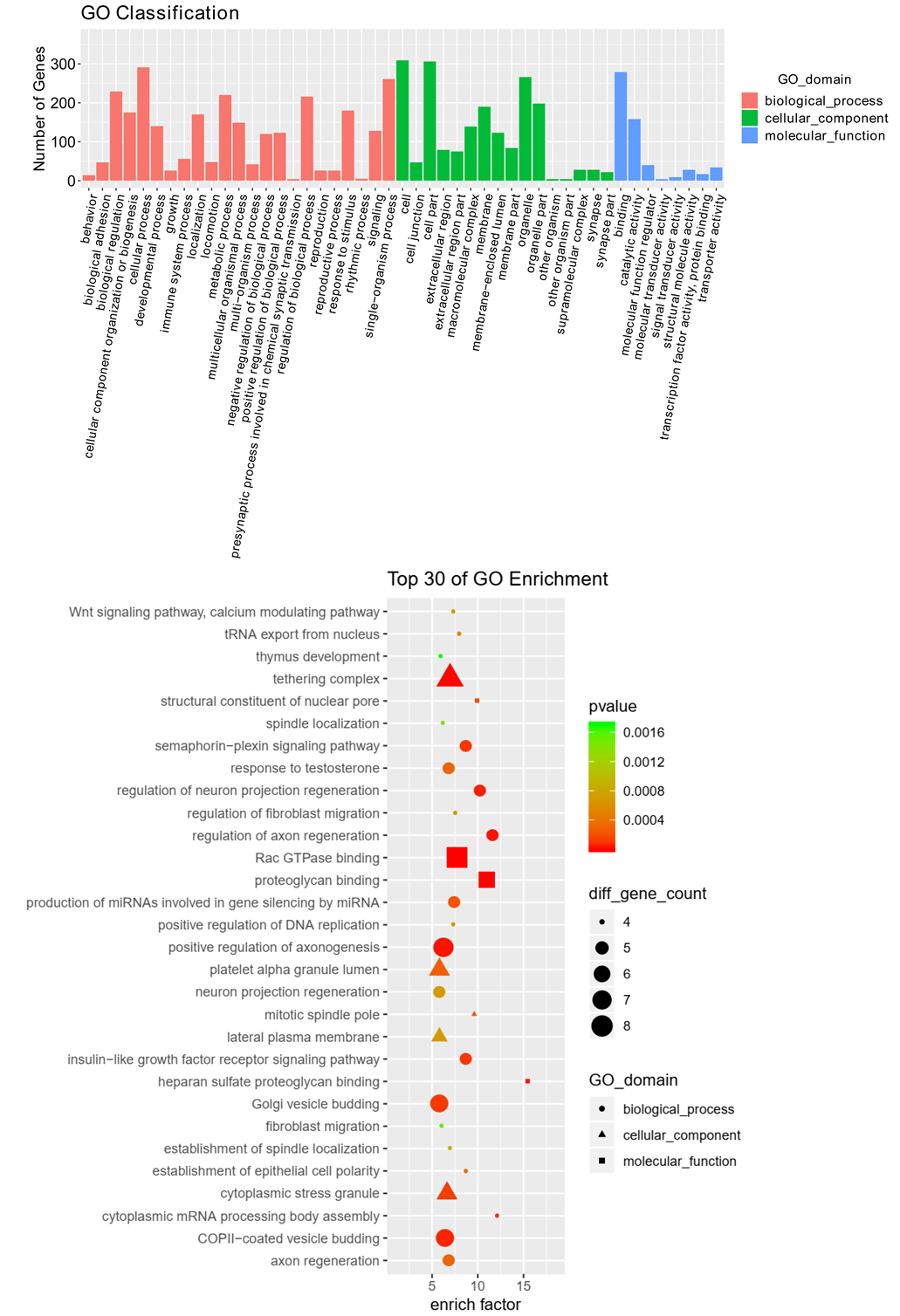
**

**Figure S5. GO analysis of** **the differential circRNA parental genes.**

**
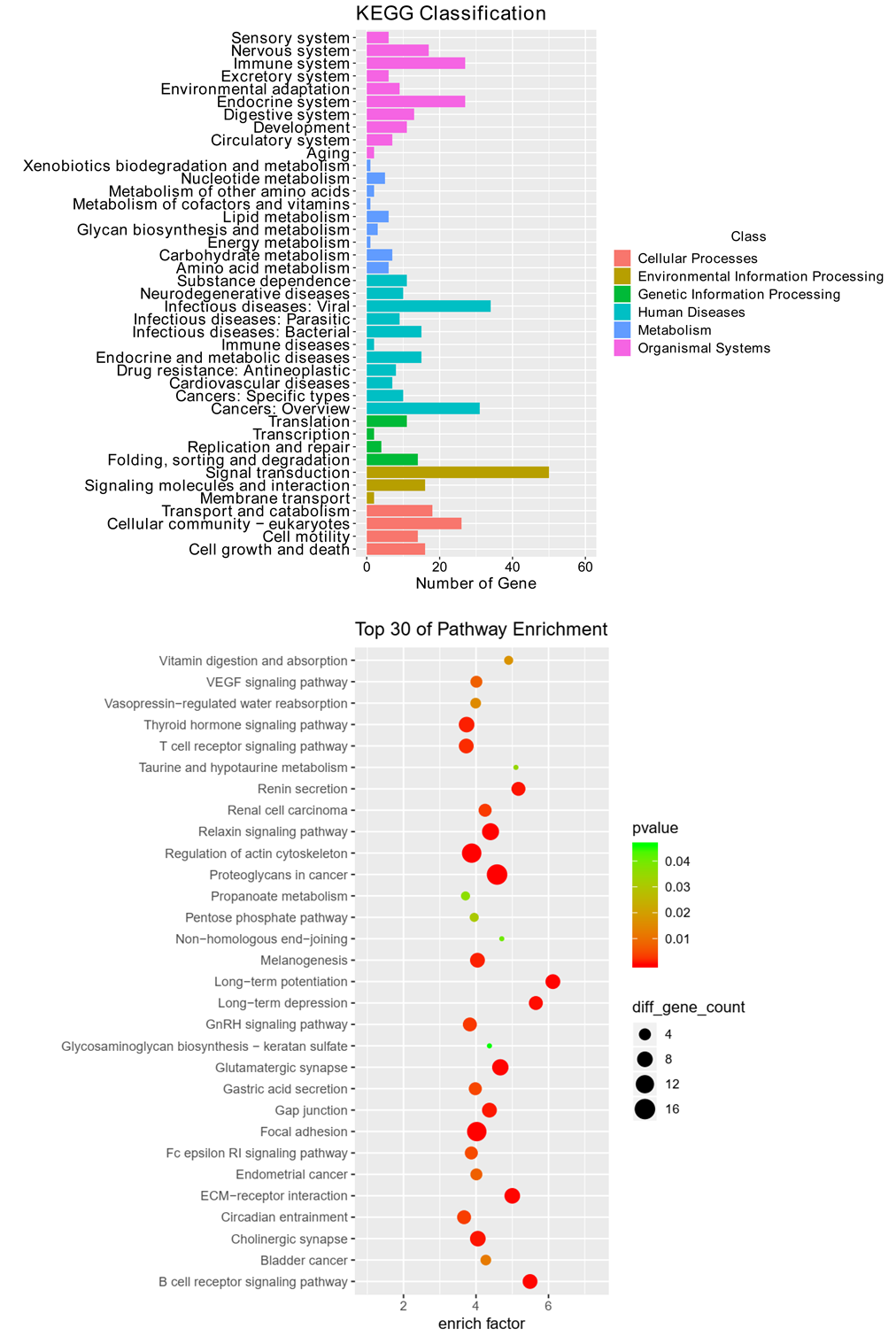
**

**Figure S6. KEGG pathway analysis of the differential circRNA parental genes.**

**
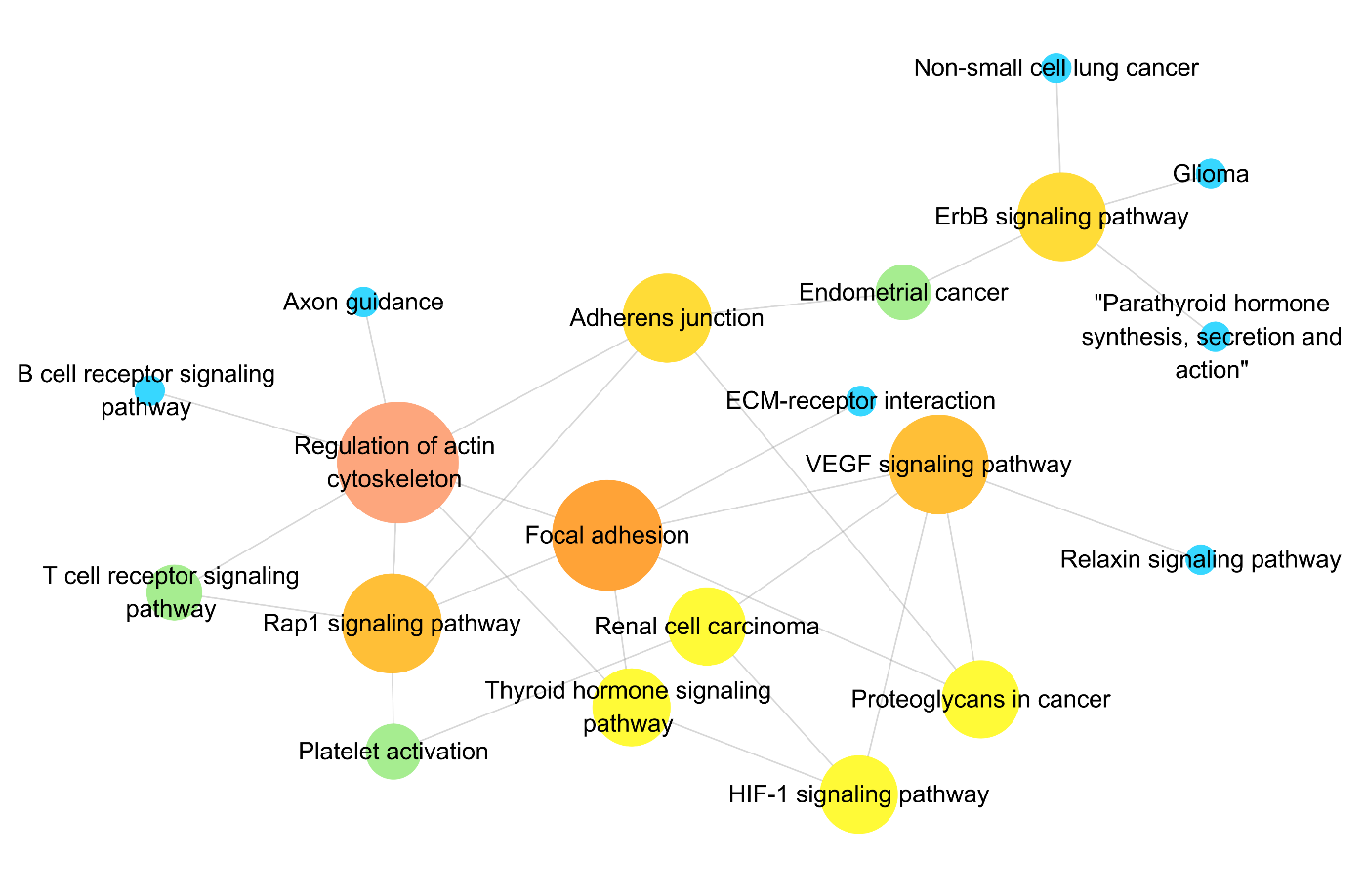
**

**Figure S7. Interaction and overlaps of the differential circRNA parental genes among significantly enriched pathways.**

**
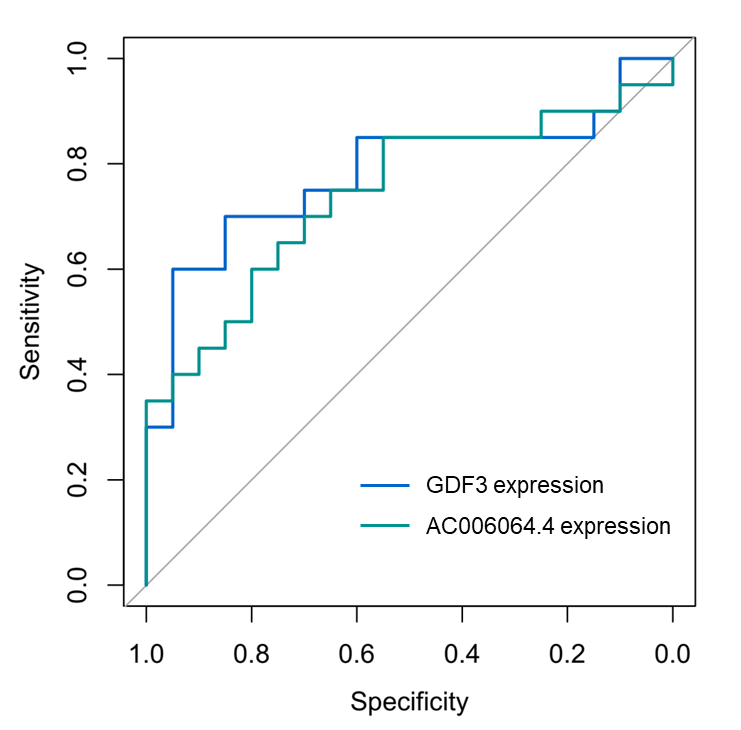
**

**Figure S8. Receiver-operating characteristic (ROC) curves of GDF3 and AC006064.4 expression in peripheral blood exosomes for prediction of GDM related macrosomia.**
